# Mechanisms Establishing TLR4-Responsive Activation States of Inflammatory Response Genes

**DOI:** 10.1371/journal.pgen.1002401

**Published:** 2011-12-08

**Authors:** Laure Escoubet-Lozach, Christopher Benner, Minna U. Kaikkonen, Jean Lozach, Sven Heinz, Nathan J. Spann, Andrea Crotti, Josh Stender, Serena Ghisletti, Donna Reichart, Christine S. Cheng, Rosa Luna, Colleen Ludka, Roman Sasik, Ivan Garcia-Bassets, Alexander Hoffmann, Shankar Subramaniam, Gary Hardiman, Michael G. Rosenfeld, Christopher K. Glass

**Affiliations:** 1 Department of Cellular and Molecular Medicine, University of California San Diego, La Jolla, California, United States of America; 2 Department of Bioengineering, University of California San Diego, La Jolla, California, United States of America; 3 A. I. Virtanen Institute, Department of Biotechnology and Molecular Medicine, University of Eastern Finland, Kuopio, Finland; 4 Department of Chemistry and Biochemistry, University of California San Diego, La Jolla, California, United States of America; 5 Biomedical Genomics Microarray Laboratory (BIOGEM), University of California San Diego, La Jolla, California, United States of America; 6 Department of Medicine, University of California San Diego, La Jolla, California, United States of America; 7 Howard Hughes Medical Institute, University of California San Diego, La Jolla, California, United States of America; Max-Planck-Institute of Immunobiology, Germany

## Abstract

Precise control of the innate immune response is required for resistance to microbial infections and maintenance of normal tissue homeostasis. Because this response involves coordinate regulation of hundreds of genes, it provides a powerful biological system to elucidate the molecular strategies that underlie signal- and time-dependent transitions of gene expression. Comprehensive genome-wide analysis of the epigenetic and transcription status of the TLR4-induced transcriptional program in macrophages suggests that Toll-like receptor 4 (TLR4)-dependent activation of nearly all immediate/early- (I/E) and late-response genes results from a sequential process in which signal-independent factors initially establish basal levels of gene expression that are then amplified by signal-dependent transcription factors. Promoters of I/E genes are distinguished from those of late genes by encoding a distinct set of signal-dependent transcription factor elements, including TATA boxes, which lead to preferential binding of TBP and basal enrichment for RNA polymerase II immediately downstream of transcriptional start sites. Global nuclear run-on (GRO) sequencing and total RNA sequencing further indicates that TLR4 signaling markedly increases the overall rates of both transcriptional initiation and the efficiency of transcriptional elongation of nearly all I/E genes, while RNA splicing is largely unaffected. Collectively, these findings reveal broadly utilized mechanisms underlying temporally distinct patterns of TLR4-dependent gene activation required for homeostasis and effective immune responses.

## Author Summary

The innate immune response is a complex biological program that is configured to allow host cells to rapidly respond to infection and tissue injury. An essential feature of this response is the sequential activation of large numbers of genes that play roles in amplification of the initial inflammatory response, exert anti-microbial activities, and initiate acquired immunity. Here, we use a combination of genome-wide approaches to characterize the basal and activated states of promoters that drive the expression of genes that are turned on at immediate/early or late times in macrophages following their stimulation with a mimetic of bacterial infection. These studies identify genetically encoded features that establish basal levels of expression and distinct temporal profiles of signal-dependent gene activation required for effective immune responses. The general features of immediate/early and late genes defined by these studies are likely to be instructive for understanding how other high-magnitude, temporally orchestrated programs of gene expression are established.

## Introduction

Precise control of gene expression in response to external cues is essential for normal development, homeostasis and immunity. In the case of the innate immune system, which provides initial protection against bacterial and viral pathogens through the utilization of germ line-encoded pattern recognition receptors [1]–[3], genes encoding proteins with antimicrobial and/or pro-inflammatory activities must be rapidly and highly induced in the presence of an infectious challenge, but maintained in a transcriptionally repressed state under normal conditions. Toll-like receptor 4 (TLR4) is a pattern recognition receptor for the lipopolysaccharide (LPS) component of gram-negative bacteria [4] and provides a widely used model system for the study of inflammatory gene expression. TLR4 signaling in macrophages activates hundreds of genes that contribute to anti-microbial activity and initiate secondary inflammatory signaling pathways that amplify acute inflammatory responses and contribute to the development of acquired immunity. TLR4 regulates gene expression of numerous transcription factors that drive inflammatory responses, including NF-κB, AP-1 and interferon regulatory factors (IRFs) [2], [3]. These factors function in a combinatorial manner to activate so-called immediate-early (I/E) genes in a protein synthesis-independent manner. In addition, feed-forward loops are built into the TLR4 response, with important examples being the production of TNFα and type I interferons. The production of type I interferons leads to secondary activation of late genes containing gamma-activated sites (GAS elements) recognized by STAT1 homodimers and genes containing interferon-stimulated response elements (ISREs) recognized by STAT1/STAT2/IRF9 complexes [5] that play roles in antimicrobial responses.

In addition to sequence-specific transcription factors, several classes of co-activator and co-repressor complexes are involved in the regulation of transcriptional responses. These complexes harbor several enzymatic functions, including nucleosome remodeling and histone modifying activities. Nucleosome remodeling activities play essential roles in controlling the accessibility of DNA regulatory elements to sequence-specific and general transcription factors [6]. Recent quantitative analysis of a cohort of 55 immediate/early and 12 late TLR4-responsive genes indicated that immediate/early and late genes lacking CpG islands were generally dependent on the activities of SWI/SNF nucleosome remodeling activities for effective gene activation [7]. In contrast, immediate/early and late promoters that were enriched for CpG islands exhibited lower levels of nucleosome occupancy and LPS-induced activation of these genes was generally independent of SWI/SNF remodeling activities. The relatively ‘open’ chromatin configuration of CpG island promoters may facilitate binding of general transcription factors required for basal expression and immediate/early transcriptional responses.

Histone modifications that include, among others, acetylation, methylation, phosphorylation and ubiquitinylation, have been proposed to represent a code that is interpreted by distinct classes of architectural and regulatory proteins that in turn determine the ability of chromatin to function as a substrate for DNA synthesis, repair and transcription [8]–[10]. Trimethylation of histone H3 on lysine 4 (H3K4me3) by Set1 in yeast [11] and orthologous members of the Mll family of histone methyltransferases in mammals [12] occurs on virtually all actively transcribed genes [13]. Genome-wide studies found that the majority of protein-encoding genes in human embryonic stem (ES) cells, liver and B cells are marked at their promoter regions by H3K4me3, and are occupied by RNA polymerase II (Pol II) [14]. Only a subset of these genes appear to generate full-length transcripts in any particular cell type, however, suggesting that H3K4 methylation confers a permissive state enabling a gene to be receptive to additional signals required for effective transcriptional elongation.

Acetylation of histones H3 and H4 by histone acetyltransferases (HATs) such as CBP, p300 and p/CAF in particular has been linked to transcriptional activation [15]–[18]. Acetylation of histone H3 at K9 and K14, for example, is required for efficient recruitment of transcription factor (TF) IID and transcriptional initiation [17]. Furthermore, acetylation of H3K9 and H3K14 has been shown to potentiate the PHD-dependent, high affinity binding of TFIID to K4-methylated H3 tails [19]. Elongation factor P-TEFb has recently been suggested to be recruited to immediate/early TLR4-reponsive promoters that have acquired H4K5/8/12Ac by the induced activities of GCN5 and PCAF [20]. Histone acetylation is antagonized by histone deacetylases (HDACs), which are thus generally associated with transcriptional repression. Emerging evidence suggests roles of NCoR/SMRT and CoREST corepressor complexes containing histone deacetylase activities in maintaining several inflammatory response genes in a repressed state under basal conditions [20]–[23].

Recent genome-wide analyses [24], [25] have shown that many genes in higher eukaryotes are subject to regulation at the point when promoter-proximal paused Pol II enters into productive elongation, as exemplified by heat shock-induced expression of heat shock genes, such as hsp70 [26], [27]. Before induction, Pol II pauses at the promoter-proximal region in a poised state, established by DSIF and NELF [27], generating only short transcripts [28]. Immediately after heat shock, P-TEFb and other positive elongation factors are recruited, leading to release of Pol II from pausing and productive mRNA synthesis. Promoter-proximal pausing may thus serve to coordinate transcriptional elongation with pre-mRNA processing. Indeed, a recent study of a subset of immediate/early and late TLR-responsive genes suggested that the immediate/early genes are marked by positive histone modifications (H3K4me3, H3K9ac), presence of Pol II and low-level expression of full-length unspliced transcripts. Upon LPS stimulation, signal-dependent acetylation of H4K5/8/12 was proposed to mediate recruitment of pTEFb, which in turn enabled Pol II elongation and mRNA processing to occur [20].

In the present studies, we use a combination of genome-wide approaches to test several features of current models and to identify additional determinants of immediate/early and late transcriptional responses to TLR4 ligation. We demonstrate that nearly all immediate/early and late TLR target genes exhibit characteristics of active genes under basal conditions regardless of CpG content and direct detectable levels of expression of mature mRNAs. We provide evidence that basal expression of I/E and late genes is initially established by signal-independent transcription factors, exemplified by PU.1, that we suggest are involved in the initial recruitment of histone-modifying machinery. We also find numerous differences in enriched sequence motifs that direct basal patterns of TBP and Pol II density, induction of H4 acetylation, and enhance transcriptional elongation that we propose collectively contribute to the distinct temporal profiles of immediate/early and late gene activation.

## Results

### TLR4-responsive genes exhibit characteristics of active genes under basal conditions

**Figure 1:**
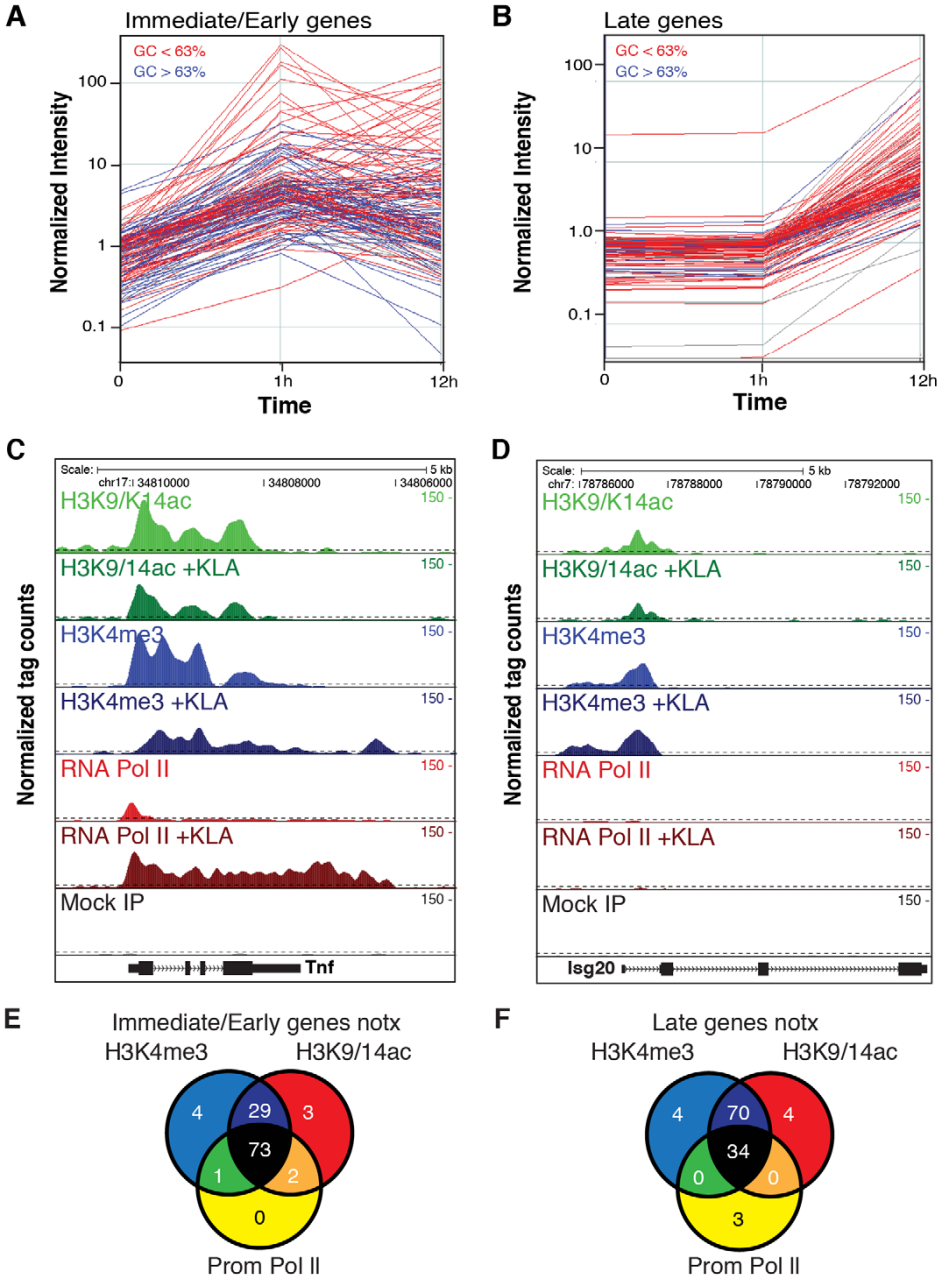
Immediate/early (I/E) and late genes exhibit characteristics of actively transcribed genes under basal conditions. A, B. Classification of I/E and late genes based on gene expression profiling of elicited peritoneal macrophages treated with the TLR4-specific agonist Kdo2 lipid A (KLA) for 0, 1 or 12 h. Values represent normalized, relative expression levels. Lines color-coded blue are represent genes exhibiting promoter GC content >63%. Lines color-coded red represent genes exhibiting promoter GC content <63%. C., D. Genome browser images of normalized tag densities for H3K4me3, H3K9/14ac and total Pol II at the *Tnf* (C) and *Isg20* (D) loci. E, F. Relationship of basal H3K4me3, H3K9/14ac and total Pol II at I/E (E) and late (F) promoters.

To study global relationships between histone modifications and TLR-dependent gene expression, we performed parallel gene expression profiling experiments and ChIP-Sequencing (ChIP-Seq) analysis in elicited mouse peritoneal macrophages (EPM) and bone marrow-derived macrophages (BMDM) treated with the highly specific TLR4 agonist, Kdo2 lipid A (KLA) [29]. ChIP-Seq was performed 1 hour after treatment with KLA or solvent control (DMSO). Similarly, mRNA was analyzed 1 and 12 hours after treatment with KLA or DMSO. Initial analysis focused on immediate early genes (I/E genes), defined as genes that were induced more than 3-fold at 1 hour after KLA treatment (Figure 1A) and late genes, defined as genes that were induced less than 1.2-fold at 1 hour but more than 4-fold at 12 hours (Figure 1B). These criteria identified 130 I/E and 120 late genes (Datasets S1 and S2), respectively. Basal levels of expression of these genes in elicited macrophages tended to be somewhat lower than in bone marrow derived macrophages, indicating that by the time of study, any potential prior activation of gene expression in peritoneal macrophages due to the elicitation procedure itself had resolved (Figure S1A, S1B). I/E genes exhibited higher promoter GC content than late genes, with a value of 0.63 GC providing maximal discrimination between the two classes (Figure S1C). Using this value as a cutoff, 43% of I/E promoters and 19% of late promoters exceeded 0.63 GC content (color coded blue in Figure 1A, 1B).

**Figure 2:**
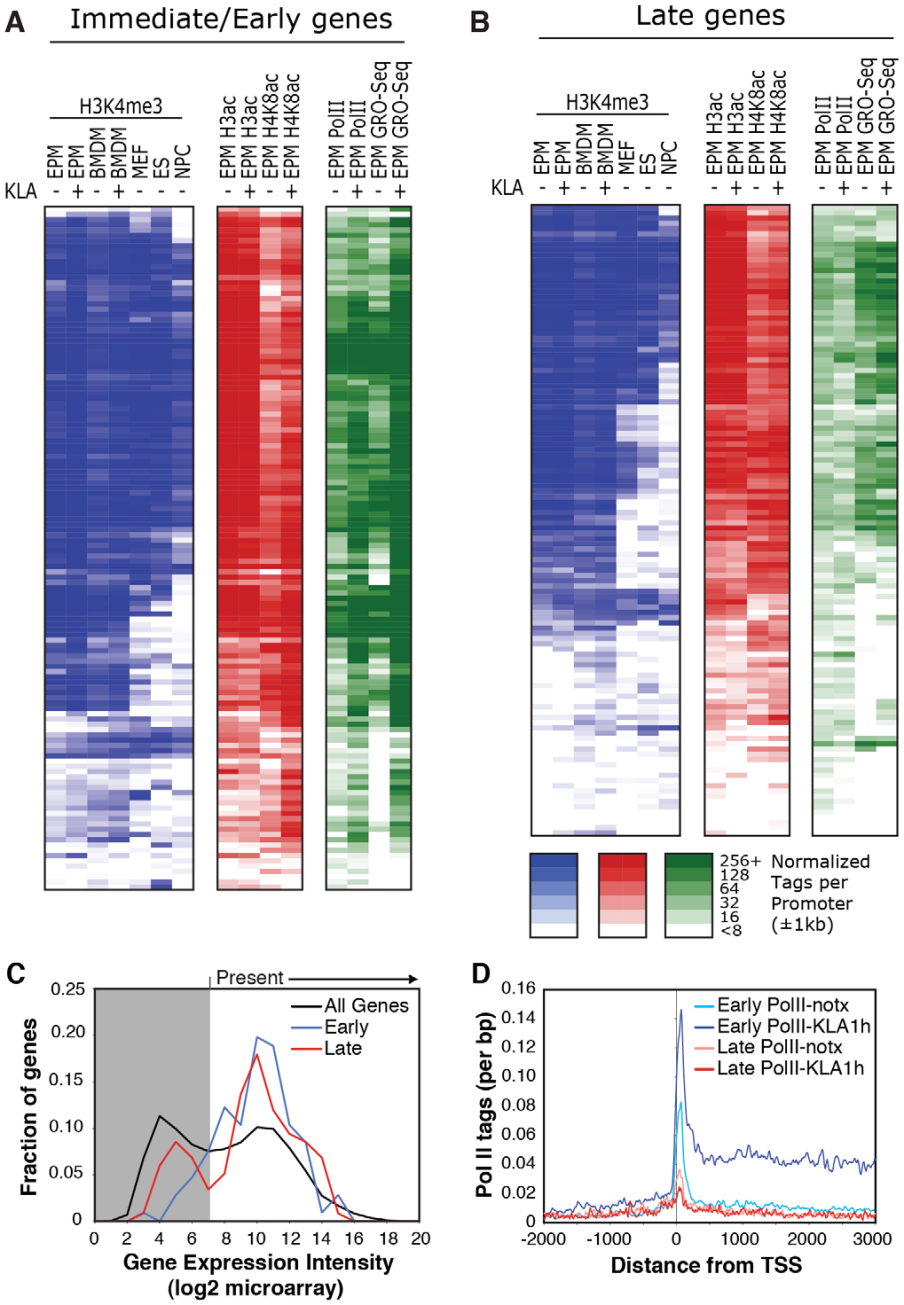
Relationships of histone modifications and RNA polymerase II at I/E and late TLR4-responsive promoters. A, B. Heat maps of normalized tag densities for the indicated histone marks, total RNA polymerase II (Pol II), or GRO-Seq. Values for murine embryonic fibroblasts (MEFs), murine embryonic fibroblasts (ES), and neuronal progenitor cells (NPCs) are based on data reported in [33]. All other lanes represent values from elicited peritoneal macrophages treated with KLA or control solvent for 1 h. C. Distribution of gene expression values as measured by DNA microarray for I/E and late TLR-responsive genes under basal conditions. Values to the right of the gray box are considered to be confidently above signal background. Similar results are found when using RNA-Seq to quantify expression levels (Figure S2C). D. Distribution of RNA polymerase II at I/E and late genes.

ChIP-Seq experiments initially evaluated trimethylation of histone H3 lysine 4 (H3K4me3), acetylation of histone H3 lysines 9 and 14 (H3K9/K14ac), and total RNA polymerase II. A total of 5–8×10^6^ unique, mappable sequence tags were collected for each antibody and treatment condition. Tags were assigned to a specific promoter if they occurred within 1 kb of the transcriptional start site (TSS) based on the global frequency distribution of these marks (). Representative genome browser images for the I/E gene *Tnf* and the late gene *Isg20* are illustrated in Figure 1C, 1D. Heat maps depicting promoter-associated tag counts for I/E and late genes in elicited macrophages are illustrated in Figure 2A and 2B, respectively, enabling visualization of tag densities for each mark in elicited macrophages on a gene-by-gene basis. Normalized tag counts for all of the data collected in this study are provided in Datasets S1 and S2.

In resting macrophages, the H3K4me3 mark was enriched over background on 89% of I/E promoters and 78% of late promoters. This pattern was observed in both elicited peritoneal macrophages and bone marrow derived macrophages (Figure 2A, 2B), indicating that the H3K4me3 mark becomes established independently of the elicitation procedure. Global alignment of the spatial distribution of H3K4me3 tags with respect to the TSS for the 130 I/E and 120 late genes under basal conditions demonstrates a bimodal distribution between +/−1 kb of the TSS, with a nadir at ∼−50 bp (Figure S2A, S2B), typical of transcriptionally active genes [30]. Although the H3K4me3 mark extends somewhat further downstream from the TSS in I/E promoters, as a group, both I/E and late promoters exhibit histone marks associated with transcriptionally active genes under basal conditions and are not distinguished by these modifications. In response to KLA, a substantial increase of H3K4me3 enrichment was observed on I/E genes around and downstream of the TSS (Figure S2B).

Acetylation of H3K9/14 exhibited a very similar distribution on both classes of promoters and was highly correlated with H3K4me3 (Figure 1E, 1F and Figure 2A, 2B). This is in line with a recent report suggesting the presence of p300/CBP on TLR4-responsive I/E genes under basal conditions [20]. Consistent with this, mapping public p300 ChIP-Seq data for resting and TLR4-activated bone marrow-derived macrophages [31] indicated that p300 was present on I/E promoters under basal conditions, with lower but detectable levels also present on late promoters (*vida infra*). Upon KLA stimulation, H3K9/14ac was largely unchanged, or trended towards a decrease (Figure 1C, 1D and Figure 2A, 2B) even though the recruitment of p300 increased (*vida infra*).

The gene expression program elicited by TLR4 agonists differs between cell types. For example, murine embryonic fibroblasts (MEFs) are capable of responding to TLR4 signaling [32], but exhibit a more limited response than macrophages. In contrast, neural progenitor cells (NPCs) and embryonic stem cells (ES) exhibit little or no response to TLR4 agonists. To assess whether these different TLR4 responses are reflected in the promoter H3K4me3 status, we compared ChIP-seq data for H3K4me3 in murine embryonic fibroblasts (MEFs), neural progenitor cells (NPCs), and embryonic stem cells (ES) from literature sources [33] with data from macrophages (Figure 2A, 2B). Consistent with the different scopes of their TLR4 response programs, only a subset of TLR4-responsive genes in macrophages are marked by H3K4me3 in MEFs, while an even smaller fractions are marked in ES cells and NPCs. These observations suggest that H3K4me3 is deposited on the promoters of inflammatory genes prior to activation in a cell type-specific manner consistent with the cell's biological functions, and is not simply a general function of promoter DNA, such as the GC-content in these regions.

Taken together, these data indicate that most I/E and late inflammatory genes in macrophages are characterized by the presence of H3K4me3 and H3K9/K14ac marks under basal conditions, exhibiting histone modifications characteristic of actively transcribed genes. These findings are in line with the observation that the basal levels of mRNA transcripts for the majority of I/E and late genes are well within the range of detection of the microarray platform or RNA sequencing approaches used for analysis (Figure 2C, ). Even for genes that were not confidently identified as present by these methods, transcripts could be identified using optimized qPCR assays (data not shown).

### Immediate/early promoters are preferentially occupied by RNA polymerase II and regulated at the level of transcriptional initiation and elongation

**Figure 3:**
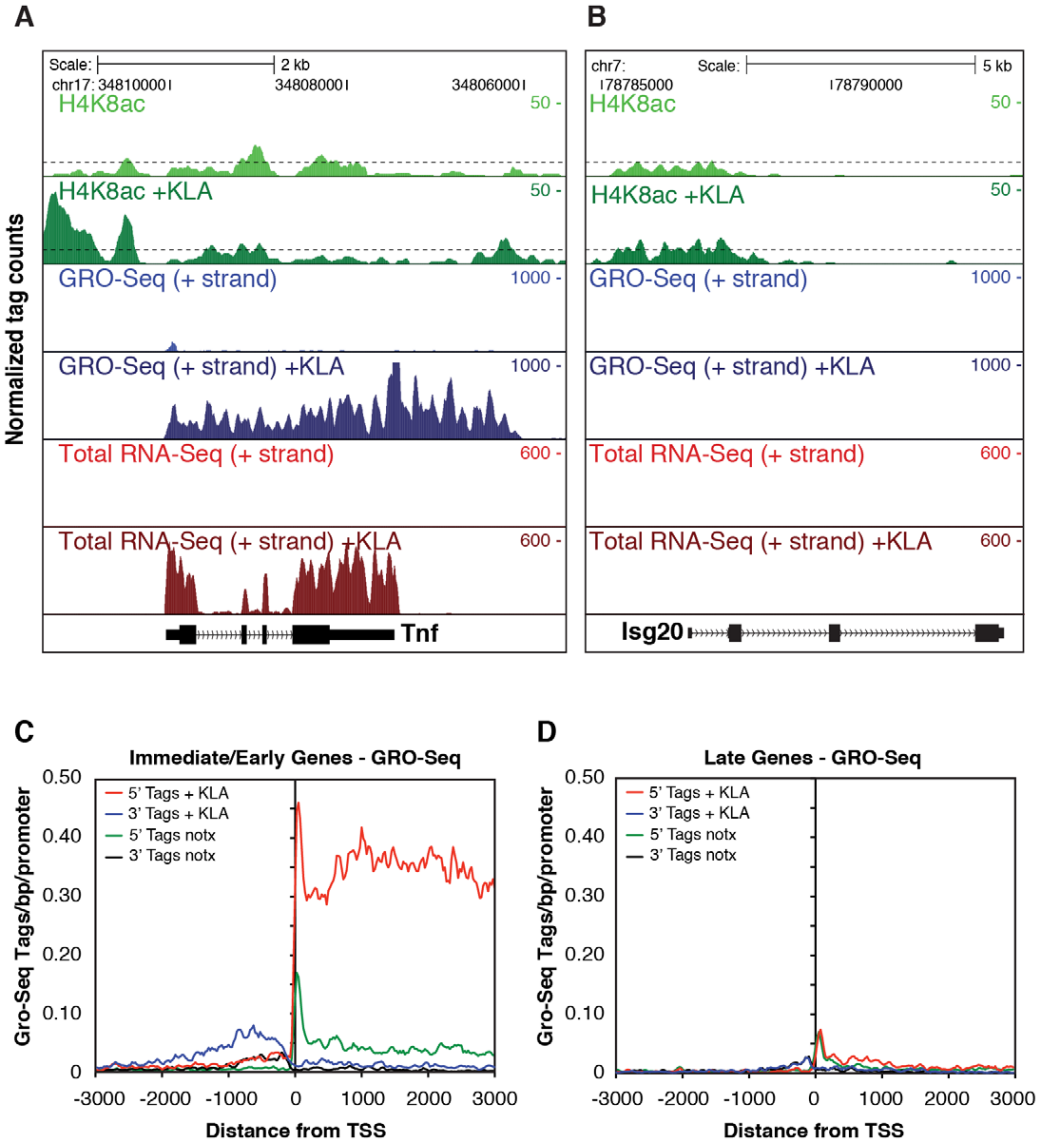
Analysis of nascent RNA transcripts (GRO-Seq), total RNA, and H4 acetylation. A., B. Genome browser images of normalized tag densities for H4K8ac and strand specific GRO-Seq, and total RNA-Seq at the *Tnf* (A) and *Isg20* (B) loci. C, D. Strand-specific distribution of GRO-Seq tag densities at I/E and late genes with KLA or control solvent for 1 h.

**Figure 4:**
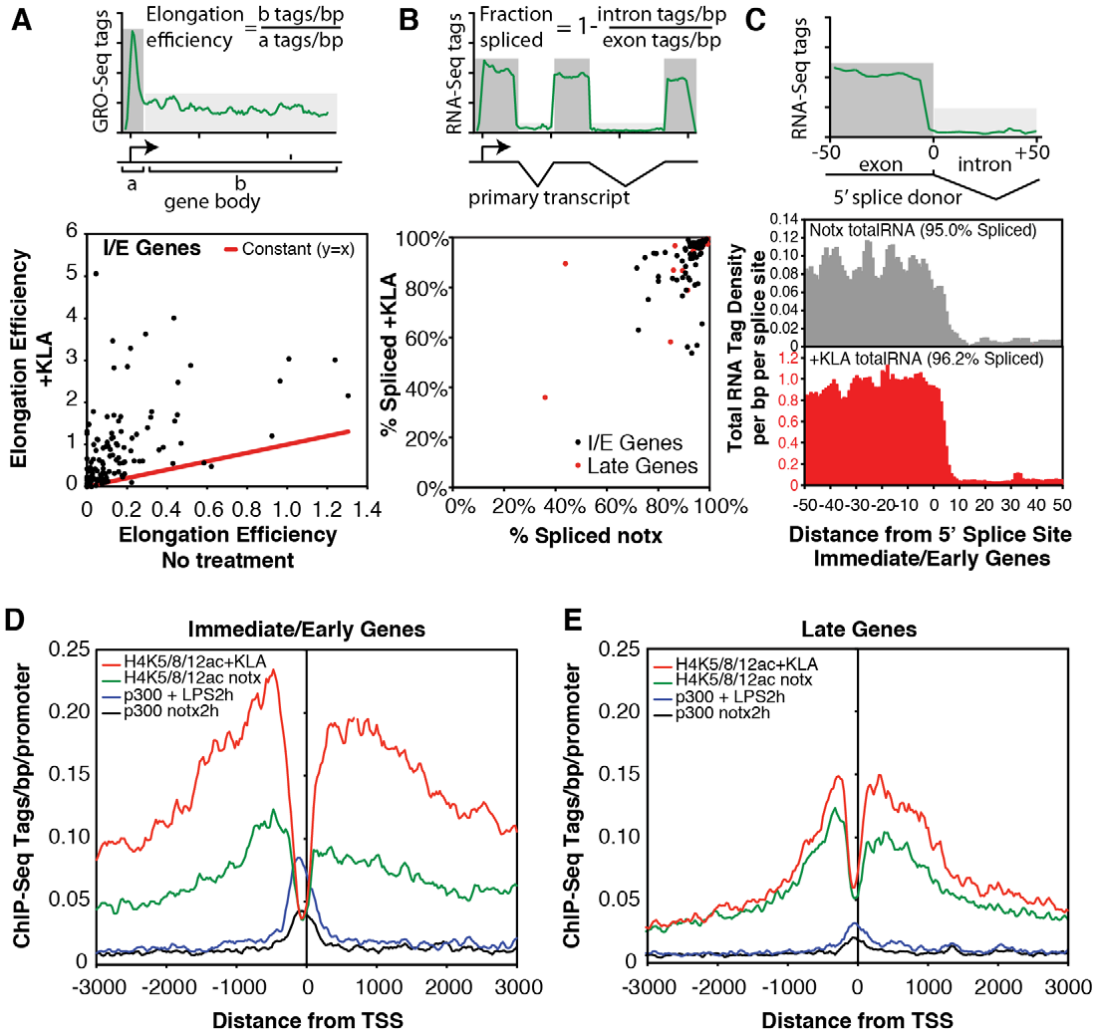
Quantification of elongation, splicing, and H4 actylation at I/E and late TLR4-responsive promoters. A. Gene-specific comparison of the elongation efficiency between control and KLA at 1 h treated samples for I/E genes. Elongation efficiency was defined as the ratio of strand-specific GRO-Seq tag density found within the gene body (+500 to +2500 bp downstream of the TSS) to the GRO-Seq tag density found at the proximal promoter (−25 bp to +175 bp). B. Gene-specific comparison of splicing efficiency between control and KLA treated total RNA-Seq samples at 1 h for I/E genes. Splicing efficiency was defined as one minus the ratio of intron RNA-Seq tag density divided by the exon RNA-Seq tag density for each gene. Only genes with intron and exon tag densities exceeding 1 read per kb were used included in this analysis. C. Visualization of tag-densities mapping across 5′ splice junctions in I/E genes. The density of the 3′ ends of total RNA-Seq tags (32 bp in length) are plotted relative to all RefSeq defined 5′ splice junctions in I/E genes. Splicing efficiency was estimated as one minus the ratio of the density of tags confidently mapping across the splice junction (3′ end from from +7 bp to +25 bp) by the density found in the exons (3′ end from −25 bp to −7 bp). D, E. Distribution of H4ac (composed of H4K5ac/H4K8ac/H4K12ac) and p300 at I/E and late genes.

Examination of Pol II enrichment profiles revealed that I/E genes but not late genes exhibit a striking enrichment for promoter-associated Pol II under basal conditions, with a peak located immediately adjacent to the first H3K4me3-positive nucleosome downstream of the transcriptional start site (Figure 1C, Figure 2D). As expected, most I/E genes showed a significant increase of Pol II after 1 hour of KLA treatment, both at the promoter region and within the transcribed region (Figure 1C, Figure 2D). These observations are in line with recent studies suggesting that many immediate/early TLR-responsive genes are regulated at the level of transcriptional elongation [20]. To further evaluate this possibility on a genome-wide scale, we performed global nuclear run-on sequencing (GRO-Seq), allowing quantification of nascent transcripts [24]. Under basal conditions, the nascent transcript density peaks near the TSS in both I/E and late genes (Figure 3A–3D), suggestive of the production of short RNA species. After KLA stimulation, the nascent transcript density near TSS was significantly increased in most I/E genes, but not in late genes, consistent with the substantial increase in Pol II occupancy. In addition, there was a disproportionately larger increase in nascent transcript density within the gene bodies of I/E genes, correlating with the marked increase in exonic RNA tags derived from total RNA sequencing (Figure 3C). (Total RNA sequencing tags in untreated cells are not visible in Figure 3A and 3B due to the use of equivalent scales for normalized tag counts). Quantification of the elongation efficiency (tag density in gene body divided by the tag density at the TSS) for all I/E genes is plotted in Figure 4A, and indicates that increased elongation efficiency is a nearly universal feature of these genes. Interestingly, we also observed an increase in the density of sense-reads upstream of the TSS, marking potential enhancer RNAs [34], and antisense TSS-associated RNAs [35] after KLA stimulation (Figure 3C).

The confirmation of a marked increase in elongation efficiency, and prior evidence that that increased processing of unspliced transcripts contributes to I/E gene expression [20], [36], prompted us to quantify the splicing of primary transcripts by sequencing total RNA. Analysis of strand specific RNA-Seq datasets of ∼6.3×10^6^ uniquely mapped reads for both untreated and KLA-treated EPMs indicated that while there were a small number of transcripts that exhibited low splicing efficiencies under basal conditions, the great majority of I/E transcripts were effectively spliced under both basal and KLA-stimulated conditions (Figure 4B, 4C).

The surprising lack of a KLA-induced change in H3K9/14 acetylation, coupled with the recent suggestion that H4K5/8/12ac provides a mark for the recruitment of P-TEFb and subequent transcriptional elongation [20], led us to evaluate the genome-wide distribution of histone H4 acetylation. Analysis of approximately 10 million unique mappable tags for each of H4K5ac, H4K8ac and H4K12ac under basal and KLA treatment conditions indicated that their overall genomic distributions were very similar (Figure S3). Gene-specific H4K5/8/12ac tag counts are indicated in Figure 2A and 2B and the aggregate tag densitites for all three marks are are illustrated in Figure 4D and 4E, indicating the presence of H4K5/8/12ac centered over both I/E and late gene promoters under basal conditions. KLA treatment for 1 h resulted in a marked increase in H4K5/8/12ac at I/E promoters, and a smaller but still significant increase at late gene promoters, concomitant with an increase of p300 occupancy. These findings corroborate on a genome-wide scale the previous findings of both basal levels of I/E and late gene expression and a KLA-dependent increase in H4K5/8/12ac that could promote transition of promoter-proximal Pol II to an elongating form at I/E promoters [20].

### Sequence characteristics of I/E and late gene promoters

**Figure 5:**
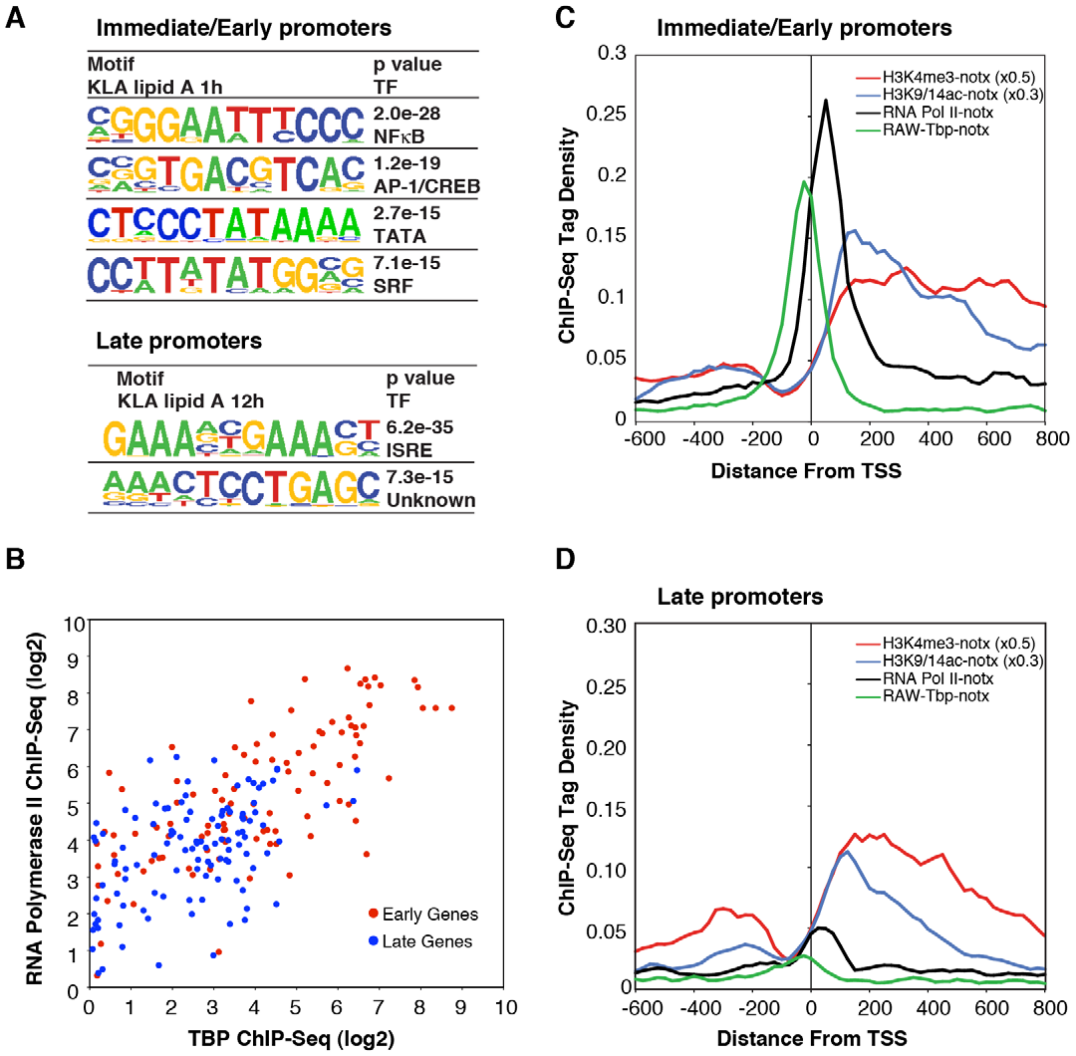
Differential use of signal-dependent transcription factors and TBP in I/E and late promoters. A. Sequence motifs identified by de novo motif analysis of the promoters of I/E and late genes. B. Scatter plots of normalized ChIP-Seq tag densities for Pol II and TBP in the interval from −100 to +250 bp from the TSS. C, D. Cumulative position-specific ChIP-Seq tag densities for H3K4me3, H3K9/14ac, Pol II and TBP determined under basal conditions for I/E (C) and late (D) genes.

The finding that I/E and late genes were both marked by H3K4me3, but that I/E genes were preferentially occupied by Pol II under basal conditions, implied the existence of sequence differences in their corresponding promoters that would determine both the kinetics of gene activation and basal levels of Pol II binding. To explore this possibility, we utilized *de novo* motif discovery methods to search for enriched sequence motifs in the proximal promoters of I/E and late genes. Motifs matching consensus binding sites for NF-κB, AP-1/CREB and SRF transcription factors were highly enriched among the promoters of I/E genes, while an interferon-stimulated response element (ISRE) and an unknown motif were the most highly enriched sequences in the late promoters (Figure 5A), consistent with prior studies [37]. In the case of I/E genes, these results are consistent with the activities of NF-κB, AP-1 and SRF factors being directly regulated by TLR4 signaling in a protein synthesis-independent manner [3]. In the case of the late genes, the enrichment for an ISRE sequence is consistent with induction of these genes being dependent on a feed-forward loop involving synthesis of type I interferons and autocrine induction IRFs.

Although these results are consistent with the different kinetics of I/E and late gene activation, they do not account for the preferential enrichment of Pol II at I/E genes. It was therefore of interest that a TATA motif was highly enriched in I/E promoters, but not in late promoters. Further position-specific motif analysis directed at the region from −35 to −20 bp from the TSS conservatively identified TATA motifs in 38% of I/E promoters, but in only 11% of late promoters. As the TATA box is recognized by TBP, which is a component of TFIID, we performed ChIP-Seq analysis of TBP under basal conditions to investigate the possibility that preferential occupancy of I/E promoters by Pol II under basal conditions was due to preferential binding by TBP. This analysis revealed a striking correlation of basal TBP occupancy with basal Pol II occupancy (Figure 5B–5D), with precise spatial relationships between TBP binding centered at −30 bp from the TSS and Pol II binding just proximal to the first downstream nucleosome marked by H3K4me3 and H3K9/14ac at +40 bp. In concert, these results suggest that the preferential recruitment of Pol II to I/E promoters under basal conditions is due to the higher frequency of TATA box-like elements in these promoters that serve to recruit and precisely position TBP.

### H3K4me3 can be established independently of signal-dependent transcription factors

**Figure 6:**
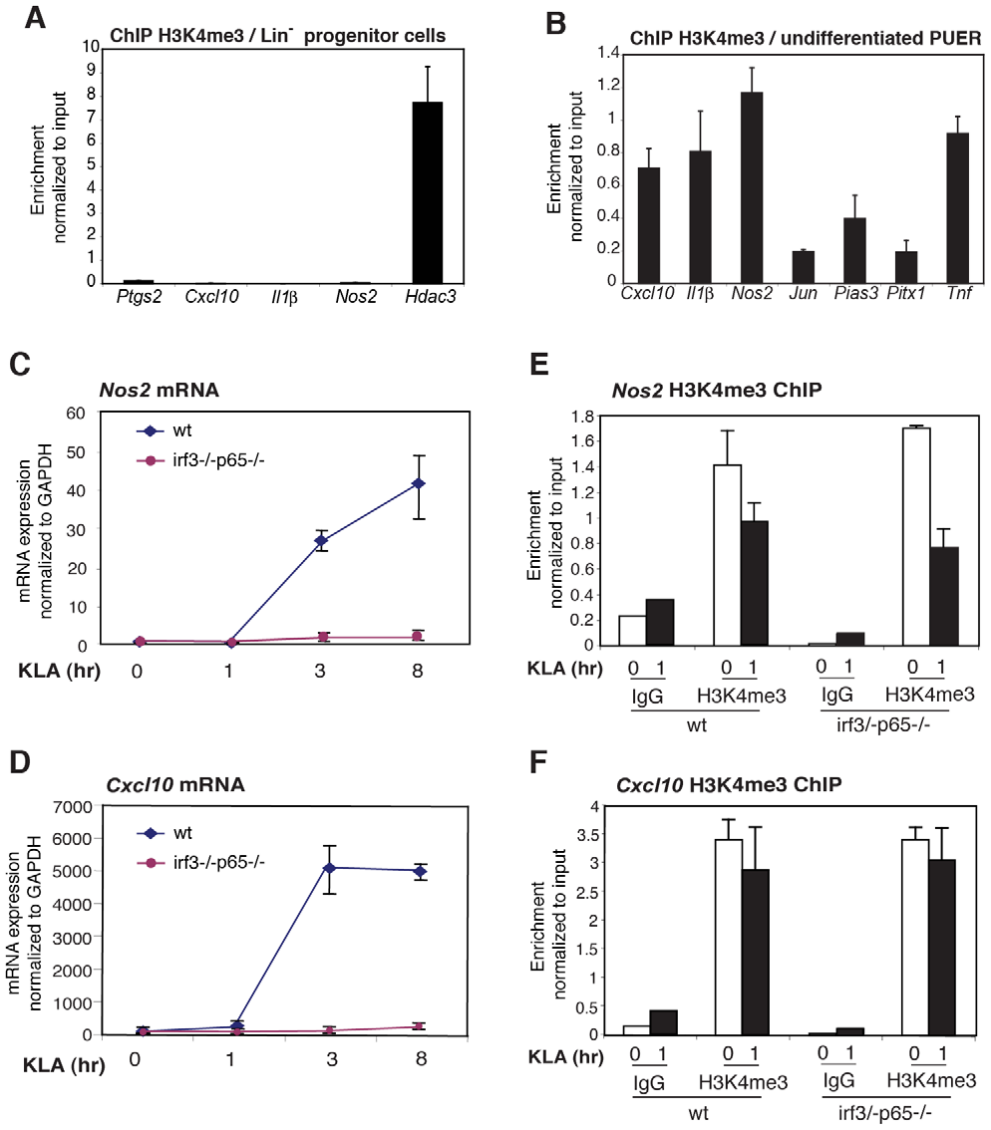
The H3K4me3 mark can be established independently of signal-dependent activators. A. The H3K4me3 mark is absent from the *Ptgs2*, *Cxcl10*, *IL1b* and *Nos2* promoters in Lin- hematopoietic progenitor cells. B. The H3K4me3 mark is present on the *Ptgs2*, *Cxcl10*, *IL1b* and *Nos2* promoters in PUER myeloid progenitor cells. C, D., KLA induction of Nos2 and Cxcl10 in MEFs requires p65 and IRF3. WT or p65/IRF3 DKO MEFs were treated for the indicated times with KLA and *Nos2* (C) and *Cxcl10* (F) mRNA levels were determined by Q-PCR. E, F., Q-PCR analysis of H3K4me3 at the *Nos2* and *Cxcl10* promoters under basal and KLA-treated conditions in WT and p65/IRF3 DKO MEFS.

As several different cell types are able to respond to TLR4 activation, but do so in a cell-specific manner, we considered the possibility that H3K4me3 is a cell-restrictive signature that marks potentially responsive target genes in a given cell type. As indicated in Figure 2A and 2B, many of the I/E and late genes that are enriched for H3K4me3 mark in basal conditions in macrophages were indeed also enriched for this mark in MEFs but more rarely in NPCs, particularly for late genes (Figure 2A, 2B). To determine whether the H3K4me3 mark becomes established in the macrophage lineage prior to hematopoietic differentiation, we used ChIP to evaluate H3K4me3 status of representative promoters in CD34^+^ lin^−^ hematopoietic stem cells. H3K4me3 was not detected on the *Ptgs2*, *Cxcl10*, *Il1b*, *Nos2* or *Pitx1* promoters in these cells, but was detected on the constitutively expressed *Hdac3* promoter (Figure 6A), indicating that H3K4 trimethylation of the TLR4-responsive promoters examined in these experiments is established during the program of macrophage differentiation. We next performed ChIP experiments to ascertain the presence of H3K4me3-positive promoters in a cell line derived from PU.1-null hematopoietic progenitor cells by transduction with a tamoxifen-inducible form of PU.1 (PUER) [38]. Analysis of the PUER cell line was performed in the absence of tamoxifen, in which the functional PU.1-estrogen receptor fusion protein is present at low levels, providing a model of an early phase of macrophage differentiation. ChIP experiments demonstrated that the *Cxcl10*, *Nos2 and Ptgs2* promoters were marked by H3K4me3 in these cells (Figure 6B).

Given that H3K4me3 is deposited during differentiation, we sought to characterize the determinants of H3K4me3 deposition. We considered two alternative mechanisms by which TLR4-responsive genes might become marked by H3K4me3 in the absence of an activating signal. One possibility is that signal-dependent activators, such as NF-κB and IRFs, might exhibit low levels of constitutive activity under basal conditions, sufficient to establish H3K4 trimethylation and low levels of gene expression. Alternatively, the H3K4me3 mark might be established by a distinct set of sequence-specific transcription factors that are alone not sufficient to confer high levels of transcriptional activity, but confer responsiveness to signal-dependent activators. To investigate the potential roles of NF-κB and IRF proteins in establishing the H3K4me3 mark, we exploited MEFs derived from wild-type embryos or embryos deficient in both p65 and IRF3. The inflammatory response of these cells is characterized by NF-κB and IRF-dependent activation of numerous chemokine-and defense response-encoding genes [32]. Consistent with this, the *Cxcl10* and *Nos2* genes exhibited a low level of expression in MEFs under basal conditions and strong induction in response to KLA, similar to results obtained in primary macrophages (Figure 6C, 6D). Furthermore, each of these genes exhibited the H3K4me3 mark under basal conditions in wild-type MEFs (Figure 6E, 66F). Significantly, the loss of p65 and IRF3 abolished TLR4-responsiveness (Figure 6C, 6D), but had no measurable impact on the presence of the H3K4me3 mark under basal conditions (Figure 6E, 6F), strongly suggesting that the poised states of these genes did not result from low levels of constitutive activity of the factors responsible for TLR4-dependent gene activation.

### Role of PU.1 in establishing TLR4 responses

Based on these results, we reasoned that signal-independent factors may be involved in establishing the poised state and thus not be enriched in promoters of TLR4-responsive genes when compared to unresponsive genes from the same cell type. To investigate this possibility, we took advantage of PU.1-null and PUER cells to evaluate potential roles of the Ets factor, PU.1, in initially establishing the H3K4me3 mark and in directing basal expression of TLR4-responsive genes. PU.1 is required for development of macrophages, neutrophils and B cells and functions as a signal-independent transcriptional activator at both promoters and enhancers [39]–[45]. Recent genome-wide location studies have demonstrated that PU.1 plays an essential role in establishing a large fraction of macrophage-specific enhancers as a consequence of collaborative binding interactions with other lineage-determining factors that include C/EBPs. These interactions enable access and function of signal dependent factors, such as nuclear receptors and NFκB. Although PU.1 primarily binds at distal genomic regions, approximately 7000 of the 60,000+ PU.1 binding sites in primary macrophages reside within 500 bp of transcriptional start sites [31], [46].

**Figure 7:**
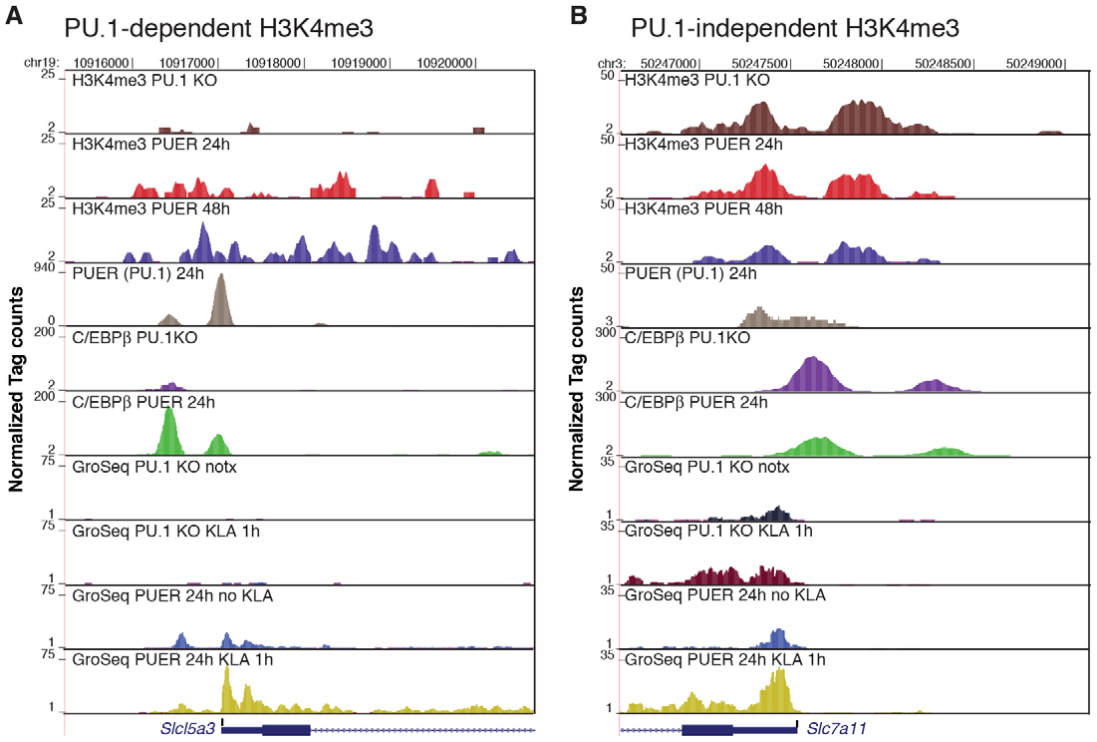
PU.1 establishes promoter H3K4me3 and basal expression required for KLA activation of a subset of TLR4-responsive genes. ChIP-Seq and GRO-Seq experiments were performed in PU.1-null hematopoietic progenitor cells (PU.1 KO) and in PUER cells treated with tamoxifen for the indicated times. Genome browser images are shown for *Slc5a3* (A) and *Slc7a11* (B). Tracks from top to bottom are H3K4me3 tags in PU.1 KO cells, H3K4me3 tags in PUER cells cultured for 24 h with tamoxifen, H3K4me3 tags in PUER cells cultured for 48 h with tamoxifen, PUER (PU.1 binding activity) tags in PUER cells cultured for 24 h with tamoxifen, C/EBPβ tags in PU.1 KO cells, C/EBPβ tags in PUER cells cultured for 24 h with tamoxifen, mRNA-strand specific GRO-Seq tags in untreated PU.1 KO cells, mRNA-strand specific GRO-Seq tags in PU.1 KO cells treated with KLA for 1 h, mRNA-strand specific GRO-Seq tags in PUER cells cultured in tamoxifen for 24 h, and mRNA-strand specific GRO-Seq tags in PUER cells cultured in tamoxifen for 24 h and treated with KLA for 1 h. ChIP-Seq data for PU.1 and C/EBPβ is from [37].

To establish the relationship between PU.1 binding, deposition of H3K4me3 and TLR4-dependent gene expression, we performed ChIP-Seq analysis for H3K4me3, PUER and C/EBPβ in PU.1-null cells and in tamoxifen-treated PUER cells. In addition, we performed GRO-Seq analysis in PU.1-null cells and tamoxifen-induced PUER cells under control conditions and following 1 h KLA treatment (Figure 7). Using normalized GRO-Seq tag counts within gene bodies as a measure of gene transcription, 170 genes were induced >3-fold after 1 h KLA treatment in tamoxifen-induced PUER cells (RPKM>0.25, FDR<10%). In the PU.1-null cells, 48 of these 170 genes were also induced >3-fold after 1 h KLA treatment, while 105 were unresponsive or induced less than 2-fold. These results indicate that the TLR4 signaling pathway is intact in PU.1-null cells, and that a substantial fraction of the TLR4-responsive genes are PU.1- dependent. Promoters that were activated by KLA in PU.1-null and PUER cells exhibited promoter H3K4me3 and measureable mRNA transcripts under basal conditions in both cell types, exemplified by *Slc7a11* and *Relb* (Figure 7B and Figure S4C).

The majority of TLR4-responsive genes that were selectively activated in PUER cells exhibited basal H3K4me3 in PU.1-null cells, but were associated with distal PU.1 binding sites (>500 bp from the TSS), consistent with roles of PU.1 in establishing signal-dependent enhancers required for gene activation by TLR4 agonists [46]. However, reconstitution of PU.1 binding also resulted in the ‘building’ of 212 new promoters as defined by induction of H3K4me3 and basal mRNA expression, 18 of which were among the set of 122 PU.1-dependent TLR4-responsive promoters. Eight of these 18 promoters were occupied by PU.1 within 500 bp of the TSS, exemplified by the *Slc15a3* (Figure 7A), *Cxcl2* and *Il1rn* promoters (Figure S4A, S4B), and the *Emr1*, *Emr4*, *SykB*, *F10* and *Ccdc88b* promoters (not shown), suggesting direct roles of PU.1 in establishing H3K4me3 and basal expression. The fraction of PU.1 promoter occupancy observed at new TLR4-responsive promoters was similar to that of the new promoters overall (82/212 new promoters exhibited PU.1 binding within 500 bp of the TSS). While the specific factors that build new TLR4-responsive promoters that do not have promoter bound PU.1 remain to be identified, these promoters are nevertheless established during macrophage differentiation by mechanisms that are independent of TLR4 signaling.

## Discussion

The present studies indicate that nearly all immediate/early and late TLR4-responsive promoters direct expression of measurable levels of mature mRNA transcripts under basal conditions and exhibit basal patterns of histone modifications (H3K4me3, H3K9/14ac and H4K5/8/12ac) that are associated with actively transcribed genes. Previous studies suggested that late genes might not exhibit these features [20], but these studies examined a relatively small set of genes at very specific genomic locations that in retrospect appear to not be fully representative of the features of late genes as a whole. In addition to directing detectable levels of basal gene expression, I/E and late gene promoters exhibited enrichment for recognition motifs for ETS, SP1 and NRF transcription factors that are typically associated with expression of housekeeping genes. By taking advantage of genetic and inducible systems, we provide evidence that signal dependent transcription factors (p65 and Irf3) are not required to establish histone marks and basal expression of TLR4-responsive promoters. In contrast, the signal-independent Ets factor PU.1 was found to contribute to the basal activation state and H3K4 trimethylation of a subset of these promoters. SP1 and other members of the Ets family, particularly Ets2, have been suggested to play analogous roles in enabling TLR4-responsiveness in macrophages [20], [47]. Notably, the use of lineage-determining factors, such as PU.1, is likely to contribute to cell-specific responses to a variety of different signaling pathways.

These findings suggest that rather than representing off/on transitions, regulation of most TLR4-reponsive genes results from a two-step process in which signal-independent factors establish basal levels of gene expression that can then be amplified by signal-dependent transcription factors, such as NF-κB, AP-1 and SRF. These signal-dependent factors can act at the promoter itself, consistent with the enrichment of their motifs within promoters, as well as at distal enhancers that may be established in a cell-specific manner [31], [46]. This general mechanism of regulation supports both the rapidly inducible, high dynamic range of gene expression necessary for the synthesis of factors that are required to amplify inflammatory responses (e.g., *Tnf*) and exert anti-microbial activities (e.g., *Nos2*), as well as the broad range of basal expression of genes that contribute to general cellular functions (e.g., Pol II elongation factors such as *Ell2*). Although it is generally considered that genes directing the expression of factors that amplify inflammatory responses must be kept in a tightly repressed state under basal conditions to prevent deleterious chronic inflammation, it is also possible that the low but detectable levels of expression of at least some of these factors serve biological functions, such as maintenance of the immune system in a state of readiness. In addition, the present studies examine populations of cells, which average out the established heterogeneity in gene expression observed at the single cell level [48].

Using a combination of genome-wide approaches, we confirm and extend the recent observation that I/E genes are enriched for a ‘paused’ form of Pol II immediately downstream of the TSS that rapidly transitions to an elongating form upon TLR4 ligation. Indeed, increased elongation efficiency in response to TLR4 ligation is observed for nearly all I/E genes. As expected, the distinct temporal responses of I/E and late genes are highly correlated with response elements for distinct sets of signal-dependent transcription factors, with I/E genes exhibiting a high degree of enrichment for NF-κB, AP-1 and SRF binding elements, and late genes exhibiting enrichment for ISRE elements. The finding that TATA boxes are preferentially enriched in I/E genes, where they are associated with highest levels of TBP and paused Pol II, provides genetic and experimental evidence for a signal-independent mechanism that enables specific promoters to become poised for rapid activation. While the TATA box can efficiently recruit the machinery for transcriptional initiation, signal-dependent elements appear necessary to recruit factors necessary for productive transcriptional elongation. As 62% of the I/E and 89% of the late promoters analyzed in these studies do not contain obvious consensus TATA motifs, other factors must also play roles in recruitment of TFIID, including signal-independent factors such as ETS proteins and SP1.

ChIP-Seq experiments also confirmed the recent observation of TLR4-induced increases in promoter-associated H4K5/8/12ac at I/E genes [20], consistent with the possibility that this modification mediates recruitment of pTEFb through Brd4. Although less pronounced, significant increases in H4K5/8/12ac were also observed at late gene promoters following 1 h of KLA treatment. Importantly, these histone modifications are present at detectable levels at nearly all I/E and late genes prior to activation, consistent with our ability to detect mature transcripts from these genes. It is notable that these marks are symmetrically distributed over the TSS and extend over relatively long distances (>3 kb). Our findings are consistent with potential roles of p300, PCAF and GCN5 HATs in establishing these marks [20], but how these HATs are recruited and the mechanisms for propagation of H4K5/8/12ac over kilobases of DNA remain to be determined. TLR4-dependent gene promoters are held in an inactive state by histone deacetylase (HDAC)-containing co-repressor complexes, which are dismissed from these promoters upon TLR4 activation [22], [49]. The rapid gains of H4K5/8/12ac on both I/E and late genes following KLA stimulation are temporally correlated with rapid, signal-dependent turnover of NCoR and SMRT complexes from both classes of promoters [22], [49], [50]. This correlation raises the possibility that H4K5/8/12 acetylation marks are substrates for NCoR/SMRT-associated HDAC3 activity, which could thereby contribute to the Pol II pausing observed under basal conditions.

The observation that the fully activated expression levels of some TLR4-responsive genes do not reach even the basal levels of expression of other TLR4-responsive genes (Figure 1A, 1B) further emphasizes the point that most TLR4-dependent gene expression represents quantitative modulation of basal levels of gene expression, rather than a qualitative transition from ‘off’ to ‘on’ at the levels of transcriptional initiation, transcriptional elongation, or post-transcriptional processing of RNA. The evolution of a hierarchy of genetic elements required for TLR4-responsiveness that are responsible for establishing active promoters (i.e. Ets, Sp1), poised transcriptional states (TATA), and signal dependent activation (i.e. kB, ISRE) provides multiple levels for gene-specific regulation. Although the present findings have focused on TLR4-dependent gene regulation, it is likely the general features of I/E and late gene promoters defined by genome-wide and gene-specific approaches will prove to be informative for understanding other complex signal-dependent programs of gene expression.

## Materials and Methods

### Accession numbers

All ChIP-Seq, RNA-Seq, GRO-Seq and Microarray data sets have been deposited in the NCBI GEO database (http://www.ncbi.nlm.nih.gov/geo/) under accession number GSE23622.

### Ethics statement

This study was performed in strict accordance with the recommendations in the Guide for the Care and Use of Laboratory Animals of the National Institutes of Health. The protocol was approved by the Institutional Animal Care and Use Committee (IACUC) of UC San Diego (Protocol Number: S01015), and every effort was made to minimize suffering.

### Cell culture

All animal work has been conducted according to relevant institutional, national and international guidelines. Peritoneal macrophages were harvested by peritoneal lavage with 10 ml ice-cold PBS 3 days after peritoneal injection of 3 ml thioglycolate. Peritoneal cells were washed once with PBS, and seeded in 10% fetal calf serum (FCS)/DMEM containing 100 U penicillin/streptomycin in tissue culture-treated petri dishes overnight. Non-adherent cells were washed off with room temperature PBS. Fresh media was applied and cells were subjected to KLA treatment or solvent control 24 h later. RAW264.7 cells were cultured in 10% FCS DMEM. Thioglycollate-elicited macrophages were isolated as previously described [51]. PU.1^−/−^ and PUER cells were propagated and the PU.1-ER fusion protein was activated with 100 nM 4-hydroxy-tamoxifen as described [46].

Mouse hematopoietic progenitor cells were obtained by enrichment of bone marrow cells for lineage-depleted cells using the StemSep murine progenitor enrichment cocktail and StemS device (Stem Cell Technologies, Vancouver, BC, Canada) as previously described [52]. Murine embryo fibroblasts were generated from E13.5 embryos and used for gene expression and ChIP studies at passage 4–6. Rela−/−Irf3−/− MEFs were generated by interbreeding single knockout strains [53], [54].

### Gene expression profiling

RNA was extracted using RNeasy kit (Qiagen, and reverse transcribed for cDNA quantification by sybergreen Q-PCR or hybridized to Agilent or Illumina mouse whole genome expression arrays. Biological triplicates were evaluated for each time point, with untreated samples serving as controls. RNA expression profiles of several genes were confirmed after reverse transcription by quantitative PCR. A combination of statistical approaches was used to define significant changes in gene expression [55], [56].

### Chromatin immunoprecipitation (ChIP)

ChIP was performed as described previously [57], with modifications. Briefly, 10–20×10^6^ cells were crosslinked in 1% Formaldehyde/PBS for 10 minutes at room temperature. The reaction was quenched by adding glycine to a final concentration of 125 mM, and the cells were centrifuged immediately (5 min, 700× g, 4°C) and washed twice with ice-cold PBS. Cells were resuspended in swelling buffer (10 mM HEPES/KOH pH 7.9, 85 mM KCl, 1 mM EDTA, 0.5% IGEPAL CA-630, 1× protease inhibitor cocktail (Roche), 1 mM PMSF) for 5 minutes. Cells were spun down and resuspended in 500 µl lysis buffer (50 mM Tris/HCl pH 7.4@20°C, 1% SDS, 0.5% Empigen BB, 10 mM EDTA, 1× protease inhibitor cocktail (Roche), 1 mM PMSF)) and chromatin was sheared to an average DNA size of 300–400 bp by administering 6 pulses of 10 seconds duration at 12 W power output with 30 seconds pause on wet ice using a Misonix 3000 sonicator. The lysate was cleared by centrifugation (5 min, 16000× g, 4°C), and 500 µl supernatant was diluted 2.5-fold with 750 µl dilution buffer (20 mM Tris/HCl pH 7.4@20°C, 100 mM NaCl, 0.5% Triton X-100, 2 mM EDTA, 1× protease inhibitor cocktail (Roche)). The diluted lysate was pre-cleared by rotating for 2 h at 4°C with 120 µl 50% CL-4B sepharose slurry (Pharmacia, Uppsala, Sweden; Before use, up to 250 µl CL-4B sepharose were washed twice with TE buffer, blocked for >30 min at room temperature with 0.5% BSA and 20 µg/ml glycogen in 1 ml TE buffer, washed twice with TE and brought up to the original volume with TE). The beads were discarded, and 1% of the supernatant were kept as ChIP input. The protein of interest was immunoprecipitated by rotating the supernatant with 2.5 µg antibody overnight at 4°C, then adding 50 µl blocked protein A-sepharose CL-4B (GE Healthcare, Piscataway, NJ, USA; protein A-sepharose CL-4B was blocked as CL-4B above, except that it was rotated overnight at 4°C) and rotating the sample for an additional 1 ½ to 2 h at 4°C. The beads were pelleted (2 min, 1000× g, 4°C), the supernatant discarded, and the beads were transferred in 400 µl wash buffer I (WB I) (20 mM Tris/HCl pH 7.4@20°C, 150 mM NaCl, 0.1% SDS, 1% Triton X-100, 2 mM EDTA) into 0.45 µm filter cartridges (Ultrafree MC, Millipore, Billerica, MA, USA), spun dry (1 min, 2200× g, 4°C), washed one more time with WB I (20 mM Tris/HCl pH 7.4@20°C, 150 mM NaCl, 0.1% SDS, 1% Triton X-100, 2 mM EDTA), and twice each with WB II (20 mM Tris/HCl pH 7.4@20°C, 500 mM NaCl, 1% Triton X-100, 2 mM EDTA), WB III (10 mM Tris/HCl pH 7.4@20°C, 250 mM LiCl, 1% IGEPAL CA-630, 1% Na-deoxycholate, 1 mM EDTA), and TE. Immunoprecipitated chromatin was eluted twice with 100 µl elution buffer each (100 mM NaHCO_3_, 1% SDS) into fresh tubes for 20 min and 10 min, respectively, eluates were pooled, the Na^+^ concentration was adjusted to 300 mM with 5 M NaCl and crosslinks were reversed overnight at 65°C in a hybridization oven. The samples were sequentially incubated at 37°C for 2 h each with 0.33 mg/ml RNase A and 0.5 mg/ml proteinase K. The DNA was isolated using the QiaQuick PCR purification kit (Qiagen, Hilden, Germany) according to the manufacturer's instructions. Antibodies against PU.1 (sc-352) and Pol II (sc-899 X) were purchased from Santa Cruz Biotech (Santa Cruz, CA, USA). Antibodies against H3K4me3 (ab8580) were from Abcam (Cambridge, MA, USA), and antibodies recognizing H4K5/6/12ac (07-327, 07-328, 07-595) and H3K9/14ac (06-599) were from Millipore (Billerica, MA, USA). Information on antibody, cell type, treatment and mapped reads is provided in Table S1.

### RNA– and GRO–Seq

Peritoneal macrophages were plated onto 15 cm plates (2×10^7^), serum starved and treated with KLA/DMSO. For RNA-Seq cells were scraped and RNA purified using TRIZOL reagent (Invitrogen, Carlsbad, CA, USA). Ribosomal RNA was depleted from DNase-treated samples using Ribominus Eukaryote Kit (Invitrogen) followed by ethanol precipitation. cDNA synthesis and library preparation then followed the protocol described in [58].

Isolation of nuclei and nuclear run-on reaction for was carried out as as described for GRO-Seq [24]. Ten million nuclei was used in the NRO-reaction. RNA was isolated using TRIZOL reagent (Invitrogen, Carlsbad, CA, USA) and treated with TURBO DNase (Ambion, Austin, TX, USA). Base hydrolysis was performed using RNA fragmentation reagents (Ambion) and the reaction was purified through p-30 RNAse-free spin column (BioRad Hercules, CA, USA). Samples were dephosphorylated with Antarctic phophatase and GRO-Seq samples were immunopurified using Anti-deoxyBrU beads (sc-32323AC, Santa Cruz). cDNA synthesis and library preparation was performed as described in [58]. Information on antibody, cell type, treatment and mapped reads is provided in Table S1.

### High-throughput sequencing and normalization

DNA from chromatin immunoprecipitation (10–50 ng) was adapter-ligated and PCR amplified according to the manufacturer's protocol (Illumina). ChIP fragments were sequenced for 36 cycles on an Illumina Genome Analyzer according to the manufacturer's instructions. The first 25 bp (32 bp for RNA-Seq/GRO-Seq) for each sequence tag returned by the Illumina Pipeline was aligned to the mm8 assembly (NCBI Build 36) using ELAND allowing up to 2 mismatches. Only tags that mapped uniquely to the genome were considered for further analysis. Analyzed ChIP-Seq experiment that were published previously can be found in the GEO database under accession numbers GSE21512 and GSE19553 or from [33]. Data analysis was performed using HOMER, a software suite for ChIP-Seq analysis and created in part to support this study. Each ChIP-Seq experiment was normalized to a total of 10^7^ uniquely mapped tags by adjusting the number of tags at each position in the genome to the correct fractional amount given the total tags mapped. This normalization was used for all downstream analysis. ChIP-Seq experiments where visualized by preparing custom tracks for the UCSC Genome browser in a manner similar to that previously described [59]. Tag densities at each promoter were determined by first adjusting the position of each tag by half of the estimated length of the isolated ChIP fragments. Tags were then summed for each promoter for gene-specific levels (e.g., Figure 2A) or at each position in the promoter to create a profile (e.g., Figure 2D). Pol II and GRO-Seq density within gene bodies was determined by adding the number of tags within the gene body defined by RefSeq and normalizing by the length of the gene. Total RNA exon and intron tag densities were calculated by adding the number of strand-specific tags found within exons and introns defined by RefSeq and dividing these totals by the lengths of these features. The implemented methods are freely available at http://biowhat.ucsd.edu/homer/.

### De novo motif discovery using promoter sequences

Motif discovery was performed using HOMER (http://biowhat.ucsd.edu/homer/, described in Heinz et al. [46]). For the purposes of this study, macrophage CAGE data (Capped Analysis of Gene Expression, Carninci et al [60]) was analyzed to accurately identify the TSS. For each gene, CAGE tags within 1 kb of the annotated TSS (RefSeq) were collected. The 100 bp region with the highest density of CAGE tags near each gene was considered the primary TSS cluster, and the single bp within that cluster with the highest number of CAGE tags was assigned as the TSS. De novo motif discovery was carried out using the sequences from −500 to +100 bp relative to the TSS.

## Supporting Information

Figure S1 A. Comparison of normalized signal intensities for I/E genes in untreated elicited and bone marrow-derived macrophages. B. Comparison of normalized signal intensities for late genes in untreated elicited and bone marrow-derived macrophages. C. Frequency distribution of GC content of I/E and late promoters.
(TIF)

Figure S2 Global and gene-specific profiles of H3K4me3, H3K9/14ac, and Pol II in resting and activated macrophages. A. Global distribution of H3K4me3 at the promoters of the indicated classes of genes aligned at the transcriptional start site under basal conditions. B. Global distribution of H3Kme3 of the indicated classes of genes in resting and KLA-stimulated (1 h) macrophages. C. Distribution of total RNA sequencing reads/kb for all genes, I/E (Early) and late genes based on ChIP-Seq reads from elicited macrophages obtained under basal conditions.
(TIF)

Figure S3 Global distribution of H4K5ac, H4K8ac and H4K12ac at E/I (A) and late (B) gene promoters under control and after 1 h KLA treatment in elicited peritoneal macrophages.
(TIF)

Figure S4 PU.1 establishes promoter H3K4me3 and basal expression required for KLA activation of a subset of TLR4-responsive genes. ChIP-Seq and GRO-Seq experiments were performed in PU.1-null hematopoietic progenitor cells (PU.1 KO) and in PUER cells treated with tamoxifen for the indicated times. Genome browser images are shown for *Cxcl2* (A), *Il1rn* (B) and *Clptm1* (C). Tracks from top to bottom are H3K4me3 tags in PU.1 KO cells, H3K4me3 tags in PUER cells cultured for 24h with tamoxifen, H3K4me3 tags in PUER cells cultured for 48 h with tamoxifen, PUER (PU.1 binding activity) tags in PUER cells cultured for 24 h with tamoxifen, C/EBPβ tags in PU.1 KO cells, C/EBPβ tags in PUER cells cultured for 24 h with tamoxifen, mRNA-strand specific GRO-Seq tags in untreated PU.1 KO cells, mRNA-strand specific GRO-Seq tags in PU.1 KO cells treated with KLA for 1 h, mRNA-strand specific GRO-Seq tags in PUER cells cultured in tamoxifen for 24 h, and mRNA-strand specific GRO-Seq tags in PUER cells cultured in tamoxifen for 24 h and treated with KLA for 1 h.
(TIF)

Table S1 Summary table for ChIP-Seq, RNA-Seq, and GRO-Seq experiments. The antibody column indicates the specific target and source of antibody for ChIP-Seq experiments, or designates the experiment as an RNA-Seq or GRO-Seq experiment. The Cell Type/Treatment column indicates the cell type and treatment conditions. The Total Mapped Reads column indicates the total mapped reads used for analysis for each experiment.
(DOC)

Dataset S1 Cumulative ChIP-Seq, RNA-Seq, GRO-Seq and microarray data for the I/E genes displayed in Figure 1A.
(XLS)

Dataset S2 Cumulative ChIP-Seq, RNA-Seq, GRO-Seq and microarray data for the Late genes displayed in Figure 1B.
(XLS)
