# Supplementary figures and images for: Mechanisms Establishing TLR4-Responsive Activation States of Inflammatory Response Genes
Source: PLoS Genet. 2011 Dec 8;7(12):e1002401. doi: 10.1371/journal.pgen.1002401 (PMC3234212; doi:10.1371/journal.pgen.1002401)

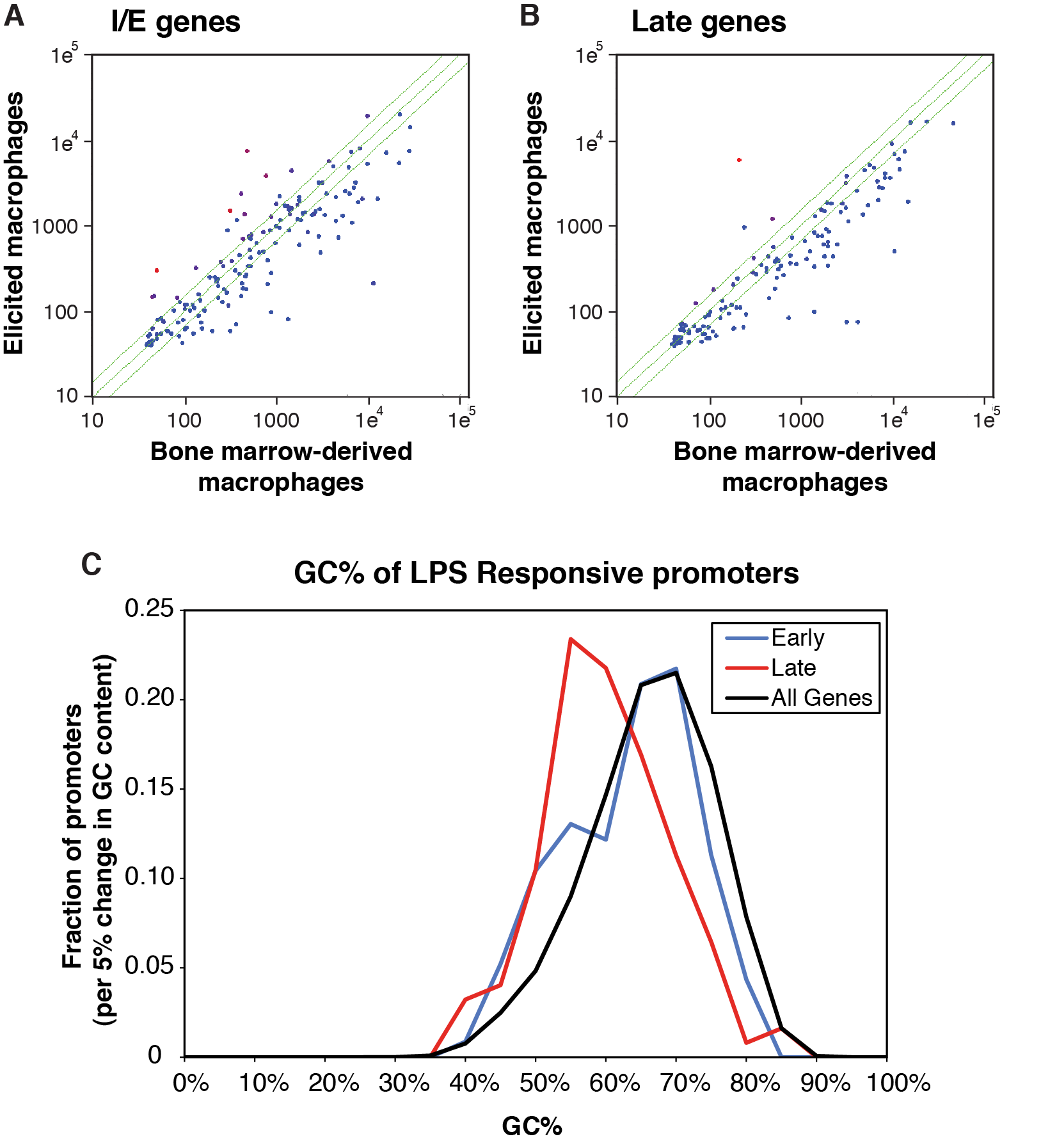

Supplement: Figure S1 — A. Comparison of normalized signal intensities for I/E genes in untreated elicited and bone marrow-derived macrophages. B. Comparison of normalized signal intensities for late genes in untreated elicited and bone marrow-derived macrophages. C. Frequency distribution of GC content of I/E and late promoters. (TIF) [file pgen.1002401.s001.tif]

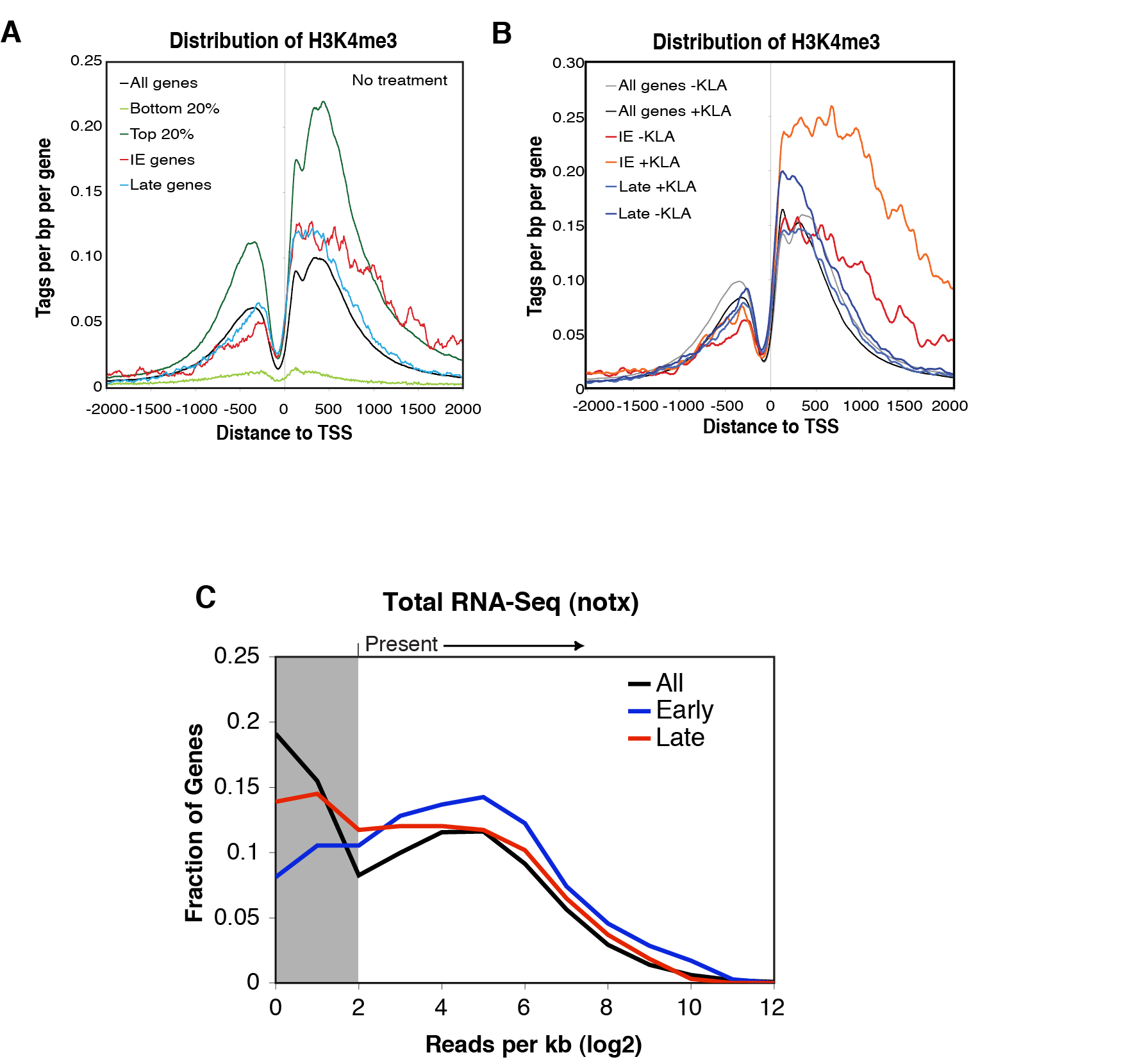

Supplement: Figure S2 — Global and gene-specific profiles of H3K4me3, H3K9/14ac, and Pol II in resting and activated macrophages. A. Global distribution of H3K4me3 at the promoters of the indicated classes of genes aligned at the transcriptional start site under basal conditions. B. Global distribution of H3Kme3 of the indicated classes of genes in resting and KLA-stimulated (1 h) macrophages. C. Distribution of total RNA sequencing reads/kb for all genes, I/E (Early) and late genes based on ChIP-Seq reads from elicited macrophages obtained under basal conditions. (TIF) [file pgen.1002401.s002.tif]

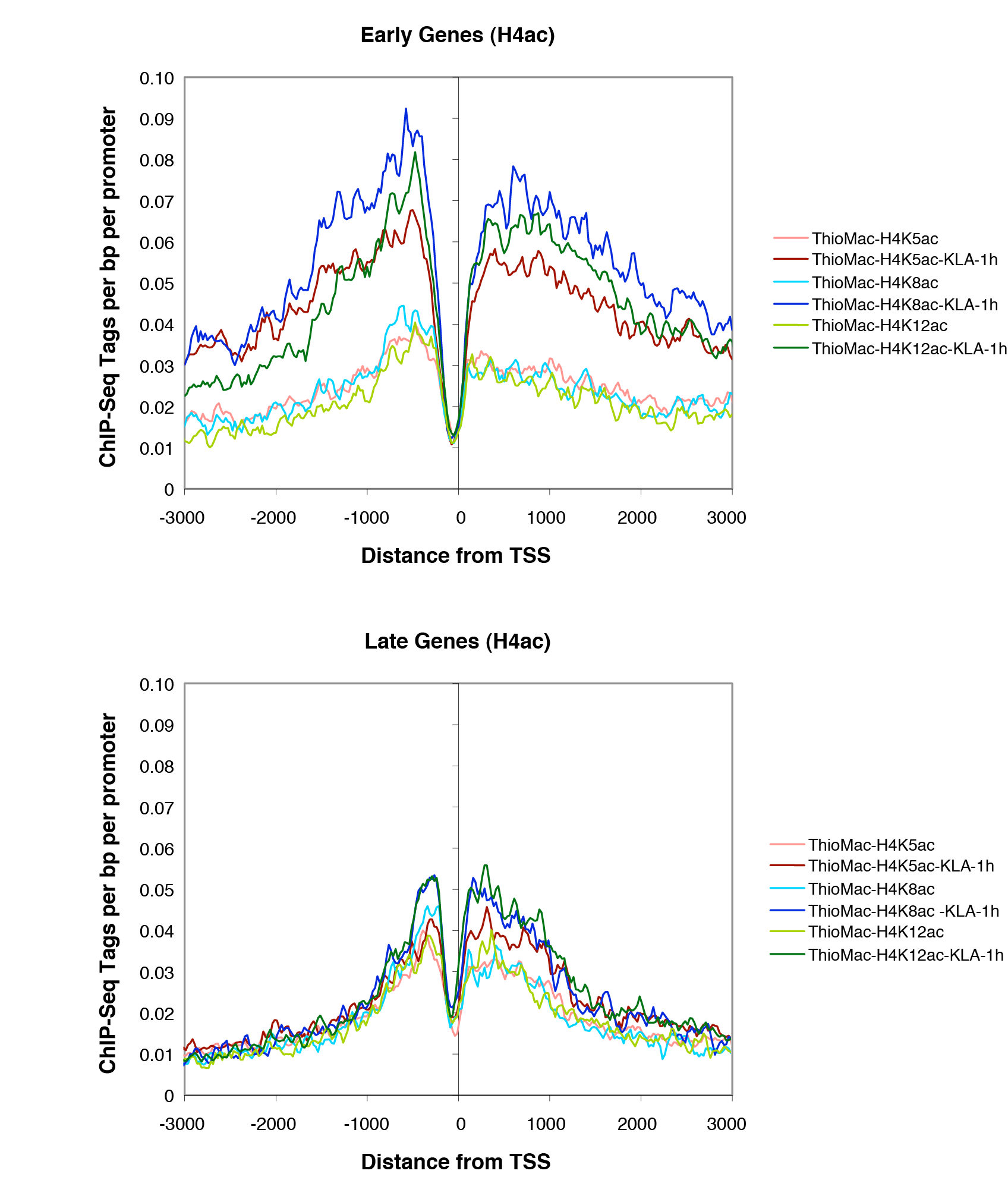

Supplement: Figure S3 — Global distribution of H4K5ac, H4K8ac and H4K12ac at E/I (A) and late (B) gene promoters under control and after 1 h KLA treatment in elicited peritoneal macrophages. (TIF) [file pgen.1002401.s003.tif]

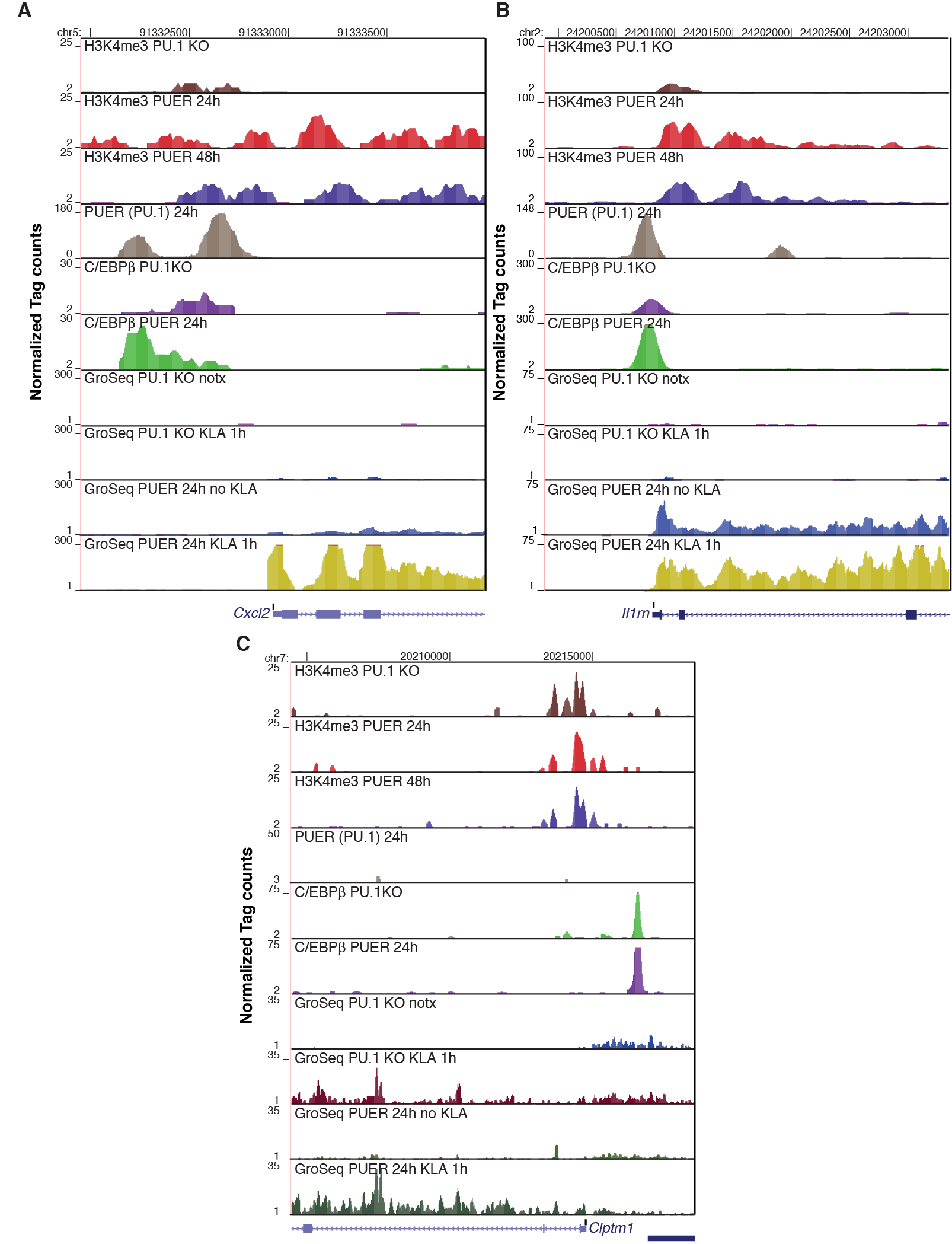

Supplement: Figure S4 — PU.1 establishes promoter H3K4me3 and basal expression required for KLA activation of a subset of TLR4-responsive genes. ChIP-Seq and GRO-Seq experiments were performed in PU.1-null hematopoietic progenitor cells (PU.1 KO) and in PUER cells treated with tamoxifen for the indicated times. Genome browser images are shown for Cxcl2 (A), Il1rn (B) and Clptm1 (C). Tracks from top to bottom are H3K4me3 tags in PU.1 KO cells, H3K4me3 tags in PUER cells cultured for 24h with tamoxifen, H3K4me3 tags in PUER cells cultured for 48 h with tamoxifen, PUER (PU.1 binding activity) tags in PUER cells cultured for 24 h with tamoxifen, C/EBPβ tags in PU.1 KO cells, C/EBPβ tags in PUER cells cultured for 24 h with tamoxifen, mRNA-strand specific GRO-Seq tags in untreated PU.1 KO cells, mRNA-strand specific GRO-Seq tags in PU.1 KO cells treated with KLA for 1 h, mRNA-strand specific GRO-Seq tags in PUER cells cultured in tamoxifen for 24 h, and mRNA-strand specific GRO-Seq tags in PUER cells cultured in tamoxifen for 24 h and treated with KLA for 1 h. (TIF) [file pgen.1002401.s004.tif]
