# Supplementary material for: Mechanisms Establishing TLR4-Responsive Activation States of Inflammatory Response Genes
Source: PLoS Genet. 2011 Dec 8;7(12):e1002401. doi: 10.1371/journal.pgen.1002401 (PMC3234212; doi:10.1371/journal.pgen.1002401)
Supplement: Table S1 — Summary table for ChIP-Seq, RNA-Seq, and GRO-Seq experiments. The antibody column indicates the specific target and source of antibody for ChIP-Seq experiments, or designates the experiment as an RNA-Seq or GRO-Seq experiment. The Cell Type/Treatment column indicates the cell type and treatment conditions. The Total Mapped Reads column indicates the total mapped reads used for analysis for each experiment. (DOC) [file pgen.1002401.s005.doc]

| Antibody | Cell Type/Treatment | Total Mapped Reads |
| --- | --- | --- |
| H3K4me3 (Abcam ab8580) | EPM notx | 6,885,937 |
| H3K4me3 (Abcam ab8580) | EPM +KLA 1h | 7,344,002 |
| H3K4me3 (Abcam ab8580) | BMDM notx | 5,263,668 |
| H3K4me3 (Abcam ab8580) | BMDM +KLA 1h | 5,391,671 |
| H3K9/14ac (Millipore 06-599) | EPM notx | 6,910,372 |
| H3K9/14ac (Millipore 06-599) | EPM +KLA 1h | 5,388,492 |
| H4K5ac (Millipore 07-327) | EPM notx | 14,566,721 |
| H4K5ac (Millipore 07-327) | EPM +KLA 1h | 14,249,884 |
| H4K8ac (Millipore 07-328) | EPM notx | 12,134,962 |
| H4K8ac (Millipore 07-328) | EPM +KLA 1h | 12,539,816 |
| H4K12ac (Millipore 07-595) | EPM notx | 12,595,859 |
| H4K12ac (Millipore 07-595) | EPM +KLA 1h | 13,877,648 |
| RNA Polymerase II (Santa Cruz sc-899 X) | EPM notx | 9,505,191 |
| RNA Polymerase II (Santa Cruz sc-899 X) | EPM +KLA 1h | 7,488,397 |
| TBP (Santa Cruz sc-204) | RAW264.7 notx | 30,443,639 |
| Total RNA-Seq | EPM notx | 6,295,771 |
| Total RNA-Seq | EPM +KLA 1h | 6,357,126 |
| GRO-Seq | EPM notx | 5,610,623 |
| GRO-Seq | EPM +KLA 1h | 5,241,956 |
